# The anatomy lesson of the SARS-CoV-2 pandemic: irreplaceable tradition (cadaver work) and new didactics of digital technology

**DOI:** 10.3325/cmj.2021.62.173

**Published:** 2021-04

**Authors:** Ivan Banovac, Vedran Katavić, Andrea Blažević, Ivana Bičanić, Ana Hladnik, Nataša Kovačić, Zdravko Petanjek

**Affiliations:** 1 Department of Anatomy and Clinical Anatomy, University of Zagreb School of Medicine, Zagreb, Croatia; 2 Croatian Institute for Brain Research and Center of Excellence for Basic, Clinical and Translational Neuroscience, University of Zagreb School of Medicine, Zagreb, Croatia; 3 Laboratory for Molecular Immunology, Croatian Institute for Brain Research, University of Zagreb School of Medicine, Zagreb, Croatia; * The first two authors contributed equally.

## Abstract

**Aim:**

To compare the efficacy of different components of online and contact anatomy classes as perceived by medical students.

**Methods:**

An anonymous course evaluation survey was conducted at the end of the academic year 2019/2020. The organization of classes due to the SARS-CoV-2 pandemic provided our students with a unique opportunity to compare online and contact classes. Students’ responses were analyzed according to the type of obtained data (ratio, ordinal, and categorical).

**Results:**

The response rate was 95.58%. Approximately 90% of students found anatomical dissection and practical work in general to be the most important aspect of teaching, which could not be replaced by online learning. During online classes, students missed the most the interaction with other students, followed by the interaction with student teaching assistants and teaching staff. Very few students found contact lectures useful, with most students reporting that they could be replaced with recorded video lectures. In contrast, recorded video lectures were perceived as extremely helpful for studying. Regular weekly quizzes were essential during online classes as they gave students adequate feedback and guided their learning process. Students greatly benefitted from additional course materials and interactive lessons, which were made easily available via e-learning platform.

**Conclusions:**

Anatomical dissection and interaction during contact classes remain the most important aspects of teaching anatomy. However, online teaching increases learning efficiency by allowing alternative learning strategies and by substituting certain components of contact classes, thus freeing up more time for practical work.

From the middle of the last century, lecturers in anatomy courses for medical students have faced two major challenges. The first has been how to incorporate the rapidly expanding new medical knowledge into the curricula. This required a reorganization of the existing curricula, and anatomy in particular was under pressure to reduce teaching hours and the student load (1-3). The second challenge has been how to modernize the teaching approach and didactically redesign the anatomy course. There has been pressure to replace cadaver work due to high expenses and high organizational demands. In many medical schools, authorities have advocated the idea that cadaver work can be replaced by other learning approaches with identical final outcomes (4). This pressure has become particularly notable in recent years and has been advocated by advancements in new digital technologies such as augmented and virtual reality (5).

Anatomy is one of the fundamental and most demanding courses in any medical school curriculum. A frequent point of discussion is how to approach teaching anatomy and facilitate students’ comprehension of difficult concepts and memorization of vast amounts of new information. Universities worldwide adopt different teaching approaches. Modern teaching usually includes a combination of teaching methods within integrated and multimodal approaches to anatomy teaching (6,7). Six techniques for anatomy education have been proposed: in-person lectures, cadaveric dissection, inspection of prosected specimens, models, radiological and living anatomy teaching, and computer-assisted learning (8). Some universities have implemented curricular changes, especially since the time allotted to anatomy education in Europe, the United States of America, and Australia has considerably declined (9). The majority of schools have switched from a completely traditional cadaver-based curriculum toward more interactive custom-made approaches that better fit the learning strategies of new generations and that appreciate technologies such as augmented and virtual reality, social networks, and imaging for a better understanding (7,10,11). Cadaver dissection, considered a gold standard for teaching anatomy (12), still remains widely used. While occasionally contested, its importance in different aspects of anatomy education has been proven by schools that returned to cadaver dissection after having temporarily abandoned it (3,13). However, meta-analyses suggest that educators should appreciate and reevaluate each instructional method in order to meet all the students’ needs, since none has so far been proven superior to any other (14).

At the University of Zagreb School of Medicine (UZSM), we teach a cadaver dissection-oriented teaching curriculum, with the use of additional teaching methods/tools, such as prosection and instructions/demonstrations on cadavers and artificial anatomical models. In recent years, we have enhanced the provided e-learning by vastly expanding the materials and activities available on our online platform for communication and teaching. We have also implemented a new, functionally oriented textbook (15,16). These changes aimed to enhance the awareness of the subject's clinical relevance and to raise the students’ active involvement in the course.

Our Department has been systematically assessing students' satisfaction with the Anatomy course through anonymous surveys (student evaluation of teaching) after the course completion. The Anatomy course is taught during two semesters in the first year of medical school. In the first semester of the academic year 2019/2020, we finished the planned curricular activities as scheduled using our usual multimethod approach. In the second semester, the SARS-CoV-2 (COVID-19) pandemic forced us to switch to exclusively online teaching for an extended period of time (17,18). Online teaching was prolonged because of the heavy damage sustained by the UZSM buildings in an earthquake that hit Zagreb on March 22, 2020 (19), immediately after the introduction of the first lock-down. We organized only a very short practical revision on cadavers and models in June, at the end of the academic year.

Such an organization of classes in the academic year 2019/2020 allowed our students to provide unique feedback about the perceived advantages and disadvantages of different components of contact and online classes. It also allowed them to evaluate the significance of these classes for meeting the anatomy course’s aims and give feedback on the overall teaching approach of the faculty. Thus, we conducted a survey with the aim of analyzing information on the efficacy of contact and online classes in covering the anatomy course material. We also analyzed how students’ success on continuous assessment during the academic year related to the way they responded to different survey questions and whether there were significant differences in those responses.

## MATERIAL AND METHODS

This study was conducted at the end of the Anatomy course (Integrated Undergraduate and Graduate Program of Studies In Medicine) at the University of Zagreb School of Medicine, as a part of the regular anonymous course evaluation survey conducted yearly.

### Curriculum

Our anatomy course is divided into three thematic blocks: A1 (General Anatomy, Anatomy of the Back and Limbs), A2 (Anatomy of the Thorax, Abdomen, and Pelvis), and A3 (Anatomy of the Head and Neck). The gross and functional anatomy of the central nervous system is covered by the Fundamentals of Neuroscience course in the second year. Each course block is divided into two parts. In part A, students learn the relevant general and systemic anatomy through lectures (mostly *ex cathedra* presentations of the course material); seminars, which include discussions in middle-sized groups; and practical work, which includes learning using prosections (previously dissected specimens) and anatomical models. In part B, students learn topographic anatomy by performing cadaver dissection themselves. After each course block, students take a partial written exam, which consists of multiple-choice and matching questions assessing their theoretical knowledge, and a partial practical exam, in which students have to name anatomical structures marked on anatomical specimens. The students who pass the partial exams are exempt from the corresponding portions of the final exam. The final exam consists of a written part, practical part, and oral part, with the final grade determined by the knowledge displayed in the oral exam.

In recent years, the Department has been gradually increasing the use of online teaching as an aid to traditional classes by adding various materials to our e-learning platform. These included engaging online activities, such as quizzes and interactive lessons covering clinically related anatomical problems (clinical cases). The Department has changed the obligatory literature to a didactically modernized textbook, which covers gross anatomy in a broader context and is functionally oriented with the aim of being more relevant to future clinical practitioners. In parallel, the Department preserved traditional classes involving practical work (on cadavers, models, and prosections) and anatomical dissection as the core of the teaching process.

In the academic year 2019/2020, due to the global SARS-CoV-2 pandemic and the extensive damage to Department of Anatomy building caused by the Zagreb earthquake, a large portion of the Anatomy course (the second half of the A2 part and the entire A3 part) was held entirely online. Online classes included pre-recorded video lectures, selected links to animations, and other freely available online material, self-evaluation quizzes, clinical cases in the form of interactive lessons, additional explanations of challenging topics, textbook elaborations, and presentation slides. During online classes, students were frequently evaluated (at least once a week) via online quizzes, through which they received bonus or malus points that were added to the results of the written partial exam. After online classes were finished, the Department organized mandatory practical (contact) classes (without dissection) as a short revision of the A2 and A3 parts, which were previously covered via online classes.

### Study design

The study was conducted via a questionnaire in the form of a course evaluation survey. The questionnaire (Supplementary material 1(Supplementary material 1)) was designed by the teaching staff of the Department of Anatomy and Clinical Anatomy and tailored to be relevant to our curriculum. This is the first time that this particular questionnaire was used at the Department of Anatomy, making this a benchmarking survey. A different, less-extensive questionnaire was used in previous years. However, due to significant changes in the curriculum structure and organization of classes (including the addition of online classes), the previous questionnaire was no longer suitable to address the relevant research questions. We plan to conduct follow-up surveys and further validate this questionnaire at the end of each subsequent academic year.

The questionnaire was made available to students online via the School’s Learning Management System (LMS) immediately after classes ended, but before the final (oral) exam was completed. Students had one week to answer the questionnaire. The answers were anonymized in the software settings, which made it impossible to connect the answers to a particular student’s identity. Before the survey was made available, we divided the students who attended our course into four groups based on the score they achieved on the partial written exams conducted throughout the academic year as a part of continuous assessment. Students with a score better than or equal to the first quartile of the generation were designated as group Q1, students with a score better than or equal to the second quartile as group Q2, students with a score better than or equal to the third quartile as group Q3, and students with a score below the third quartile as group Q4. The questionnaire results were then also filtered based on the defined groups. The results remained anonymous even when forming groups as the software prohibits connecting the survey responses to a particular student’s identity.

The questionnaire consisted of 70 questions, 27 of which were relevant to the research presented in this study (assessment of the efficacy of different types of classes in covering the anatomy course material) (Supplementary Table 1(Supplementary table 1)).

In most questions, students had to choose a number between 1 and 5 that best represented their agreement with a given statement (Supplementary Table 2(Supplementary table 2)), with 1 representing complete disagreement and 5 representing complete agreement. In some questions, students either had to write a short answer (the number of hours spent studying anatomy) or choose multiple answers from a predefined list of options. To be able to submit the survey, students had to answer every question.

### Quantitative data analysis

Responses to the questions in which students chose a number (grade) representing their agreement with a statement were analyzed as ordinal data, and the median and mean were calculated from the grades given by students.

Responses to the questions in which students chose one or more answers from a predetermined list of possible answers were analyzed as categorical data, and frequencies for each answer were presented as the percentage of students who chose a particular answer.

Responses to the questions in which students wrote the number of hours spent studying anatomy were analyzed as ratio data (ie, continuous variables), and the mean and standard deviation were calculated for each answer. The normality of the distribution was evaluated with GraphPad Prism’s in-built software, which runs four normality tests.

For comparisons between the student groups with different partial exam scores (Q1, Q2, Q3, and Q4) we used one-way ANOVA with Tukey’s *post hoc* test for ratio data and the Kruskal-Wallis test (one-way ANOVA on ranks) with the Dunn *post hoc *test for ordinal data. In the *post hoc* tests, a *P*-value of <0.05 was considered significant. For parametric tests, 95% confidence intervals were also calculated. Cross-tabulation was used to analyze the differences between groups for categorical data.

To compare the responses given by the same students to different questions, the paired Wilcoxon rank test was used for ordinal data and the paired *t*-test for ratio data. For both tests, a *P*-value of <0.05 was considered significant. Only the questions involving the comparison of online and direct classes were analyzed in this way (responses to Question 5 were compared to Question 6, and responses to Question 8 were compared to Question 9). Quantitative data analysis was performed with GraphPad Prism, version 8.3.0 (GraphPad Software, La Jolla, CA, USA).

## RESULTS

### Survey target population size, statistical relevance, and response rate

The target population for this survey were the first-year medical students at the UZSM who actively participated in the Anatomy course (both in direct classes and online activities) throughout the entire course duration. Five enrolled students discontinued their studies before the course ended and were not included in the survey, nor were they considered to be the survey target population. The total number of active medical students in the academic year 2019/2020 was 340. With a target population size of 340 students, the required number of responses for this type of survey would be 181 for a confidence level of 95% and a margin of error of 5% (20). The survey was available to all active first-year medical students, and 325 filled out the questionnaire, resulting in a response rate of 95.58%. Due to the high response rate and a sufficient number of responses, the study results can be considered an accurate representation of the entire first-year medical student population and are statistically relevant.

The most relevant questions of the survey are shown in Table 1 and are numbered from 1 to 9, while a complete overview of all the questions analyzed in this study is shown in Supplementary Table 1(Supplementary table 1), where the questions are labeled from S1 to S27.

**Table 1:** The most relevant questions in the survey and responses given by students

		Frequency distribution (%) for grades chosen as responses to statement												
Questions		1	2		3	4		5	1 and 2		4 and 5	Mean grade		Median grade
**1**	**I regularly used the available online material in learning.**	0.00	3.37		15.34	33.44		47.85	3.37		81.29	4.26		4
**2**	**I have put a great deal of effort into learning the required exam material.**	0.00	1.23		7.98	24.54		66.26	1.23		90.80	4.56		5
**3**	**Frequent quizzes (both during regular and online classes) contributed to my successfully mastering the required exam material.**	3.68	6.44		11.66	24.23		53.99	10.12		78.22	4.18		5
**4**	**Online classes are a significant and useful addition to contact classes, but cannot replace it.**	1.84	4.91		13.50	28.83		50.92	6.75		79.75	4.22		5
**5**	**During contact classes, I could easily assess my knowledge and progress at any given time.**	1.23	4.91		20.25	36.20		37.42	6.14		73.62	4.04		4
**6**	**During online classes, I could easily assess my knowledge and progress at any given time.**	6.75	21.17		32.52	26.99		12.58	27.92		39.57	3.17		3
**7**	**This course was too challenging.**	9.20	25.46		40.18	21.78		3.37	34.66		25.15	2.85		3
		**Mean response ± standard deviation (h/d)**												
		**All groups**		**Group Q1**			**Group Q2**			**Group Q3**			**Group Q4**	
**8**	**During contact classes I spent, on average, the following amount of hours per day studying Anatomy:**	4.09 ± 1.52		4.40 ± 1.57			4.29 ± 1.55			3.91 ± 1.40			3.87 ± 1.40	
**9**	**During online classes I spent, on average, the following amount of hours per day studying Anatomy:**	5.56 ± 2.46		6.13 ± 2.36			5.75 ± 2.59			5.42 ± 2.36			5.16 ± 2.39	

### Evaluation of students’ personal engagement and involvement in the course

Overall, students assessed their personal engagement in the course as high – 95.71% of students stated that they attended contact classes regularly (grades 4 and 5 on Question S1) and 81.29% of students stated that they accessed the School’s LMS regularly (grades 4 and 5 on Question 1). Both values were also confirmed by the Department’s official records. Students’ preparation for contact classes was also evaluated – students prepared most for practical work (89.26% gave grades 4 and 5 on Question S5) and least for lectures (only 49.08% gave grades 4 and 5 on Question S3), while they prepared moderately for seminars (73.32% gave grades 4 and 5 on Question S4). A total of 90.49% of students (grades 4 and 5 on Question 2) claimed to have put a high level of effort into learning the required material – this is in line with the fact that 93.50% of students successfully passed the final exam.

### Evaluation of the course material, course organization, and course structure

The most relevant findings from the student’s evaluation of the course material, organization, and structure were as follows: 78.22% of students agreed (grades 4 and 5) that regular online quizzes helped them learn the required course material, 63.50% agreed (grades 4 and 5) that learning outcomes helped them learn the required course material, 87.12% agreed (grades 4 and 5) that the required course material was important for the medical profession, and only 25.15% (grades 4 and 5) considered the course to be too difficult (Table 1 and Supplementary Table 1(Supplementary table 1)). Furthermore, 73.62% of students agreed (grades 4 and 5) that classes (both contact and online) helped them cover the required course material, with only 8.59% (grades 1 and 2) disagreeing with this statement (Question S12).

**Figure 1:**
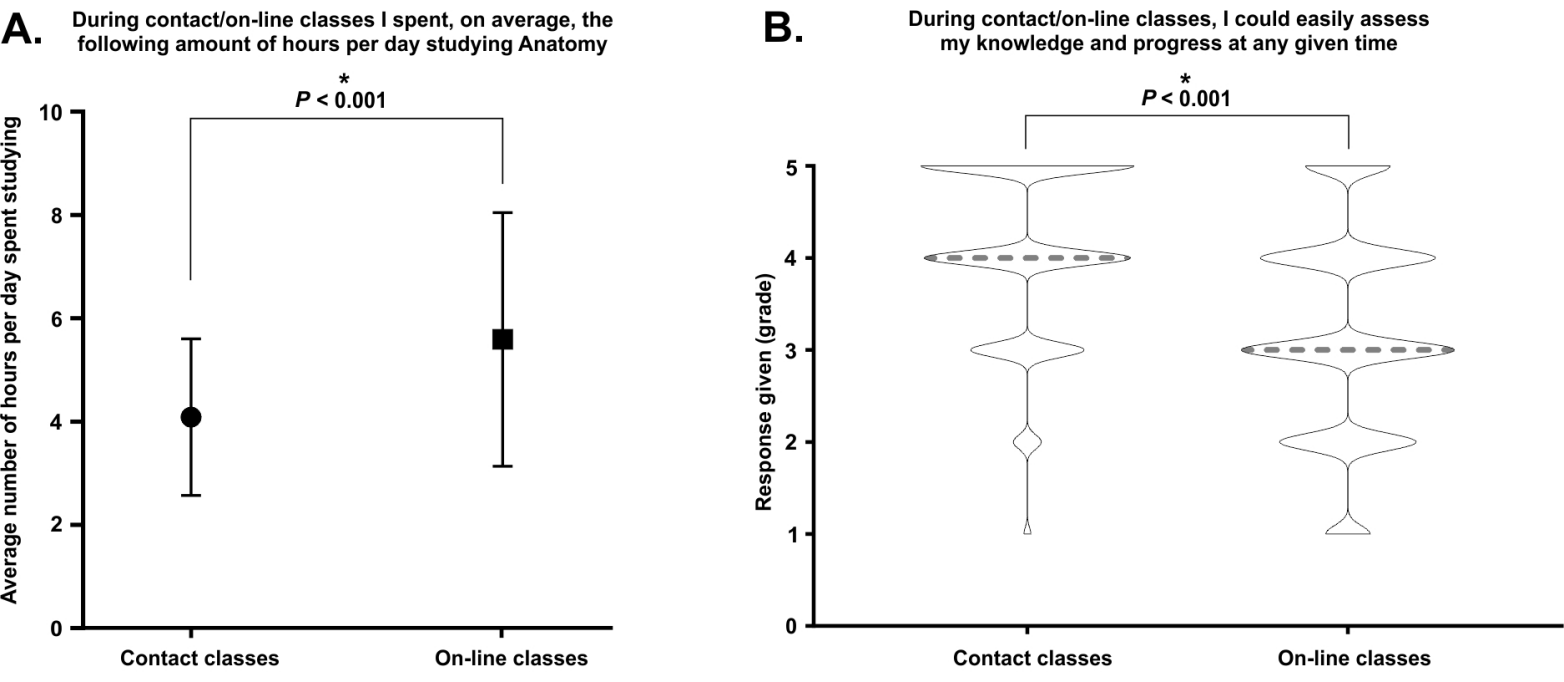
Comparison of students' responses to questions about contact and online classes. (**A**) Comparison of responses to questions in which students had to write the average number of hours per day that they spent studying anatomy during contact classes (Question 8) and during online classes (Question 9). Markers (black circle and square) represent the mean, error bars represent the standard deviation, and the *P*-value is shown on the plot (paired *t* test). (**B**) Violin plots showing a smoothed distribution of students' responses to the question in which students evaluated how easily they could assess their knowledge and progress during contact classes (Question 5) and during online classes (Question 6). The dotted line in the violin plots represents the median of the responses to each question. The size of the bulge in a violin plot is proportional to the frequency of students who chose the grade displayed on the y-axis. The *P*-value is shown on the plot (paired Wilcoxon rank test).

Students estimated that they spent on average 4.09 ± 1.52 h/day studying anatomy during contact classes and 5.59 ± 2.46 h/day during online classes (Questions 8 and 9). The difference in the responses to these questions was significant (*P* < 0.001) (Figure 1A).

A total of 73.44% of students agreed or mostly agreed (grades 4 and 5) that they could easily assess their knowledge and progress during contact classes, while only 6.14% disagreed or mostly disagreed (grades 1 and 2) with this statement (Question 5). Only 39.57% agreed or mostly agreed that they could easily assess their knowledge and progress during online classes (Question 6), while 27.92% disagreed or mostly disagreed. The difference in the responses between these two questions was also significant (*P* < 0.001) (Figure 1B).

### Evaluation of student teaching assistants

Students most favorably assessed student teaching assistants (STAs), with only 0.31% disagreeing (grade 2 on Question S19, no student gave a grade 1 on this question) with the statement that the STAs performed well. More than two thirds (69.33%) of students regarded practical work with STAs (practicing on cadavers and prosection with STAs outside regular classes defined by the curriculum) as one of the three segments of contact classes from which they benefited the most in preparing the course material and 65.64% of students rated it as one of the three segments of contact classes that they missed the most during online classes. Furthermore, 72.09% of students expressed that the interaction with STAs was one of the three aspects of contact classes they missed the most. There were no significant differences between the student groups regarding STA evaluation.

### Evaluation of contact classes

**Figure 2:**
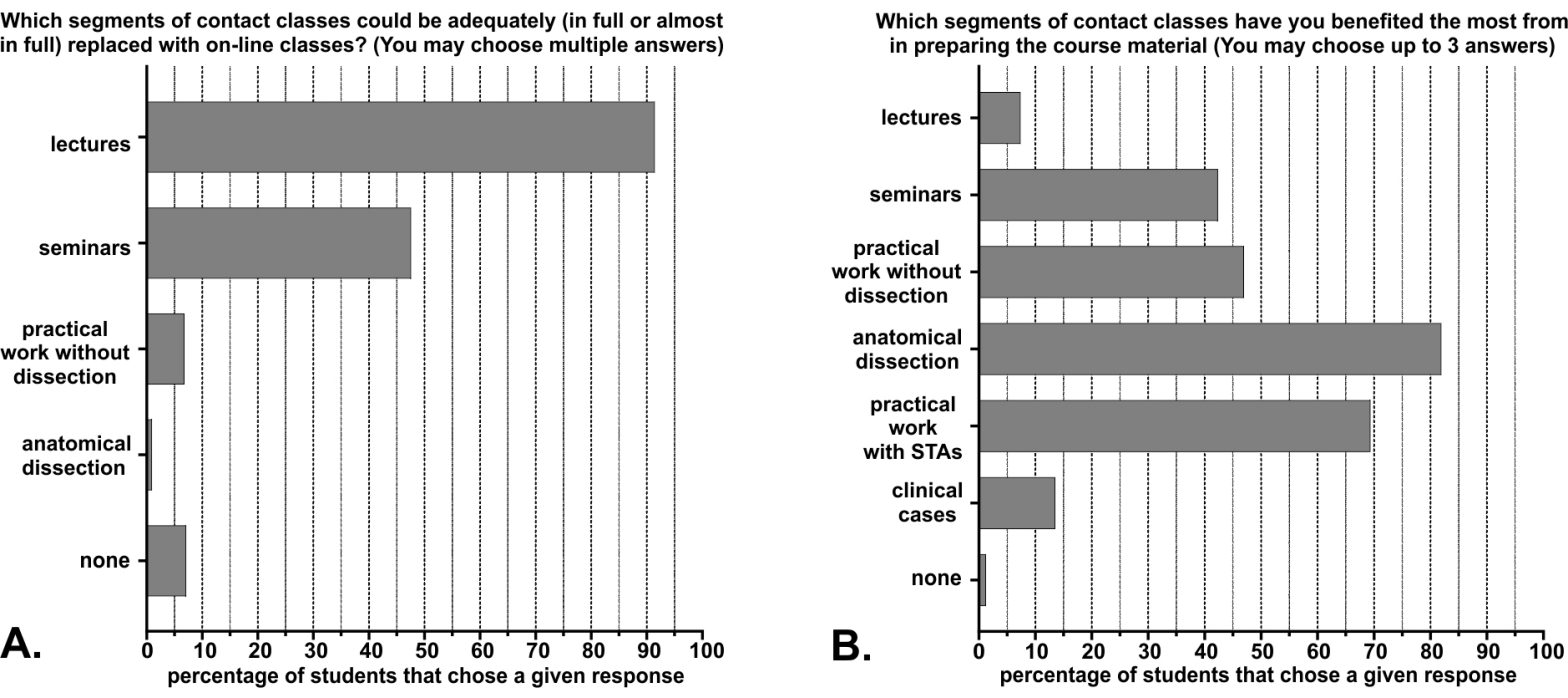
Bar graphs showing the response frequencies for the following questions: (**A**) Which types of contact classes could be adequately replaced with online classes? (Question S23); (**B**) Which types of contact classes did you benefit from the most in preparing the course material? (Question S24).

A total of 91.41% of students singled out contact lectures to be suitable for replacement with pre-recorded online video lectures (Question S23, students could choose any number of answers out of five options). Furthermore, 47.55% of students found seminars to be suitable for replacement with either webinars or recorded video lectures. Only 6.75% of students thought that regular practical work (demonstrations on models, cadavers, and prosections) could be replaced with online materials and as few as 0.92% thought the same for anatomical dissection. Only 7.06% of students felt that nothing could be replaced by online classes (Figure 2A).

Students singled out anatomical dissection as a segment of contact classes that aided them the most in covering the course material, with 81.90% of responses (Question S24, students could choose up to three answers out of seven options). Practical work with STAs was the second most important segment (69.33%), followed by regular practical work (demonstrations on models, cadavers and prosections) with the teaching staff (46.93%). Only 7.36% of students found traditional lectures to be important for learning the course material, while only 1.23% found none of the contact classes to be helpful in this regard (Figure 2B).

### Evaluation of online classes

When asked which segments of online classes aided them the most in covering the course material (Question S25, students could choose up to three answers out of 12 options), 60.74% of students chose pre-recorded video lectures and 54.29% chose regular mandatory online quizzes. Students also recognized the importance of various additional text materials, such as hand-outs (48.47%), additional explanations of challenging topics (37.42%), and textbook elaborations (explanations of inconsistencies in the textbook and further elaborations of topics the students assessed as unclear in the text) (27.91%). A substantial portion of students also appreciated the possibility to evaluate online quizzes (30.37%) by analyzing the questions and correct answers. Presentation slides and the use of the forum were viewed as less essential (chosen by only 6.44% and 3.37% of students, respectively). As with contact classes, only 1.23% found none of the online materials helpful in learning the course material (Supplementary Figure 1A(Supplementary figure 1)).

The three segments of contact classes that students missed the most during online classes (Questions S26, students could choose up to three answers out of seven options) were anatomical dissection (81.90%), practical work with STAs (65.64%), and practical work with teachers without dissection (49.08%) (Supplementary Figure 1B(Supplementary figure 1)).

The aspects of contact teaching that students missed the most during online classes to adequately prepare the course material (Question S27, students could choose up to three answers), were practical work (81.60%), the interaction with STAs (72.09%), and the interaction with fellow students (60.74%). Fewer students missed face-to-face interaction with the teaching staff (51.11%) (Supplementary Figure 1C(Supplementary figure 1)).

Out of a series of statements comparing online and contact classes (Questions S8-S11), students agreed most with the following: “On-line classes are a significant and useful addition to contact classes but cannot replace them,” with 79.75% of students agreeing or mostly agreeing.

### Response comparison between different student groups

**Figure 3:**
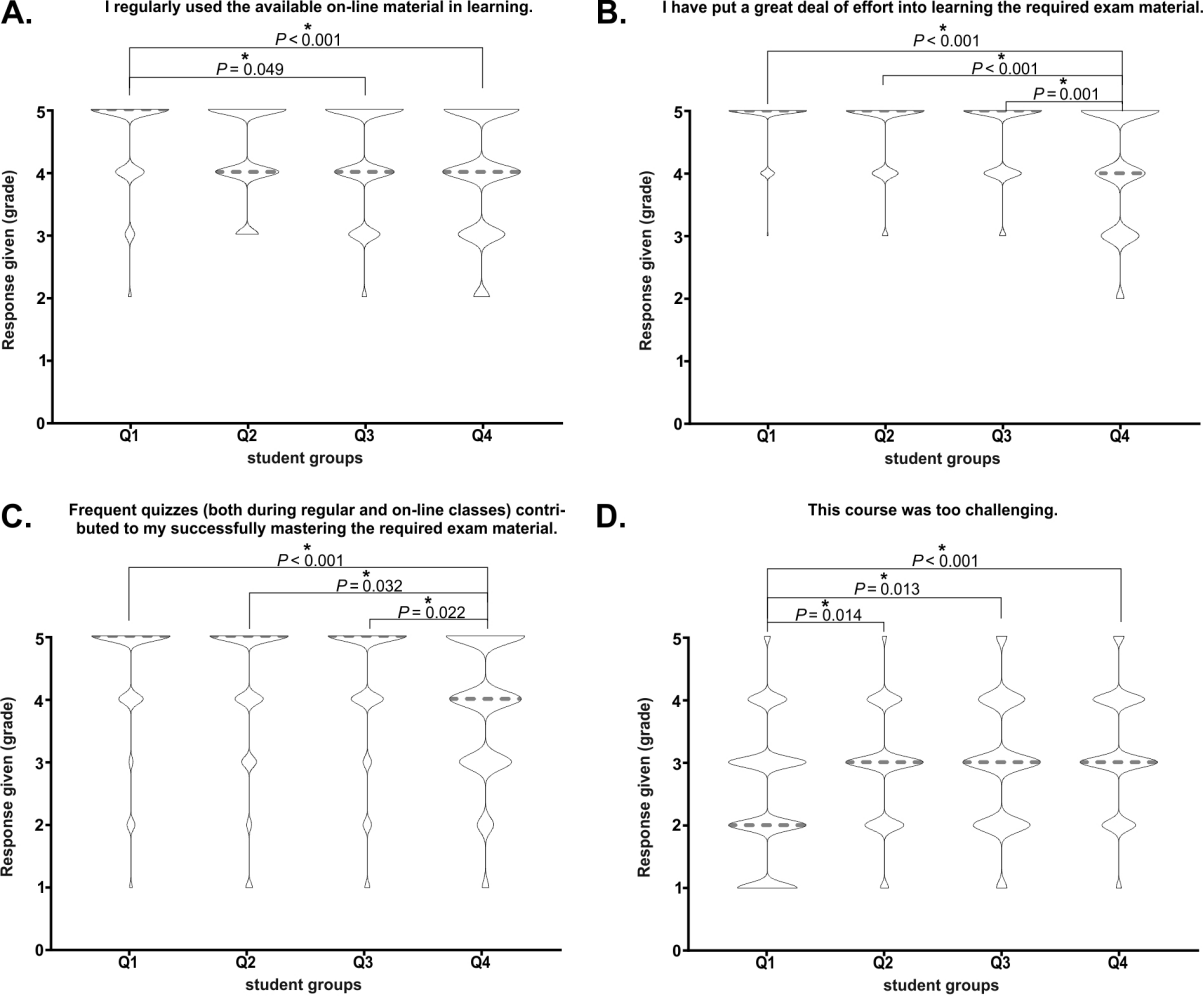
Violin plots showing a smoothed distribution of responses by students from different groups (divided into quartiles based on their written partial exam score during continuous assessment: Q1, Q2, Q3 and Q4) to the questions in which significant differences between groups were found. The dotted line in the violin plots represents the median of the responses to each question. The size of the bulge in a violin plot is proportional to the frequency of students who chose the grade displayed on the y-axis. Significant differences are marked on the plots (Kruskal-Wallis test with the Dunn *post hoc *test). The different panels show responses to the questions pertaining to (**A**) the use of online material in learning (Question 1), (**B**) the amount of effort students put into learning the course material (Question 2), (**C**) the usefulness of frequent quizzes for mastering the course material (Question 3), and (**D**) the difficulty of the course (Question 7).

For most questions in which students had to choose a grade representing their agreement with a given statement, there were no significant differences between student groups (based on their successfulness on partial written exams: Q1, Q2, Q3, and Q4). The exception were questions 1, 2, 3, 7, S3, S4, and S5, the responses to which were significantly different between certain groups (Figure 3 and Supplementary Figure 2(Supplementary figure 2)).

Students from the Q1 group spent on average the most time studying, while students from the Q4 group spent on average the least time studying. This was slightly more pronounced during online classes (Table 1). However, due to the large variability within the groups, none of the differences between groups was significant (Supplementary Figure 3(Supplementary figure 3)).

**Figure 4:**
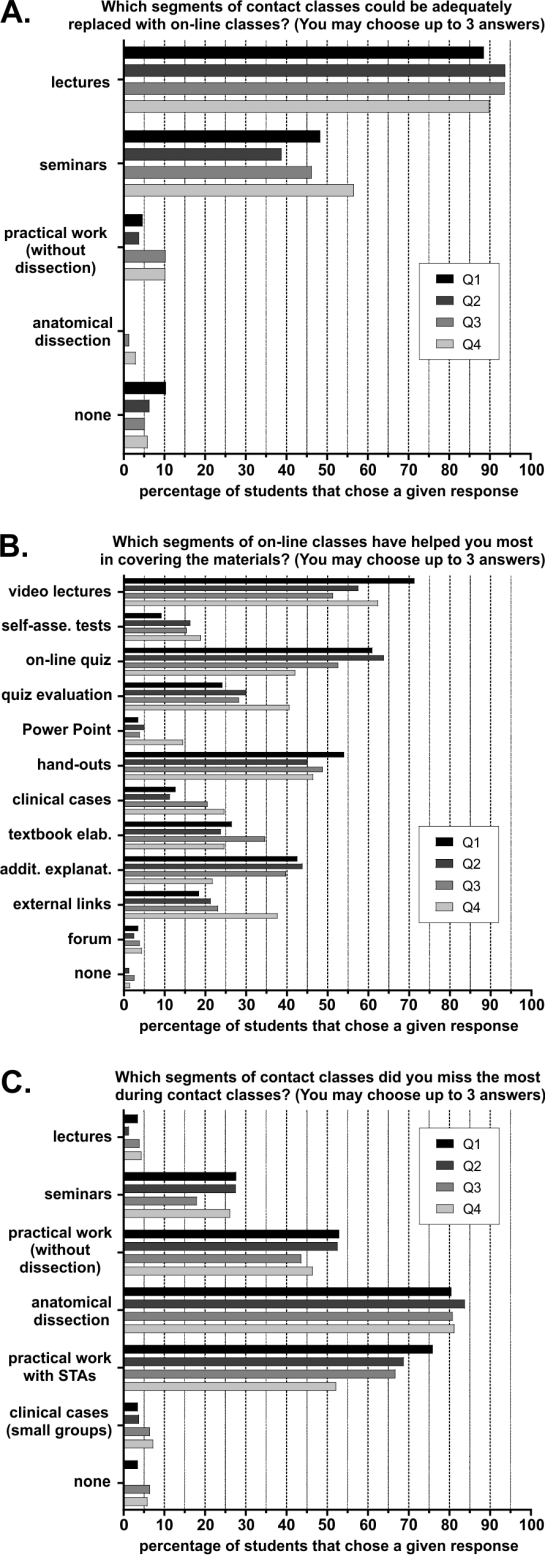
Bar graphs showing the results of the crosstabulation analysis. Students' responses are shown by groups (divided into quartiles based on their written partial exam score during continuous assessment: Q1, Q2, Q3 and Q4) to the questions on: (**A**) which types of contact classes could be adequately replaced with online classes (Question S23), (**B**) which segments of online classes helped students the most in preparing the course material (Question S25), and (**C**) which types of contact classes students missed the most during online classes (Question S26).

For questions in which students had to choose the answers from a predefined list, the crosstabulation analysis revealed that most groups responded similarly, but there were some noticeable differences in certain responses (Figure 4) – primarily in the way different groups responded to the questions pertaining to online classes.

When asked which segments of online classes helped them the most in preparing the course material (Figure 4A), students from the Q1 group noticeably more often chose pre-recorded video lectures (71.26%) than all other groups (57.50%, 51.28%, and 62.32% for Q2, Q3, and Q4, respectively). Students from the Q4 group considered mandatory online quizzes as less beneficial (42.03%) than other groups did (60.92%, 63.75%, and 52.56% for Q2, Q3, and Q4 respectively), however, they also considered the evaluations of the online quizzes more beneficial (40.58% vs 24.14%, 30.00% and 28.1% for Q2, Q3, and Q4, respectively). Furthermore, students from the Q4 group considered self-assessment tests, online clinical cases, and links to animations more beneficial than any other group, while they also considered additional explanations of challenging topics less beneficial than any other group (Figure 4B).

When asked which aspects of contact teaching they missed the most during online classes (Figure 4C), students from the Q1 group mostly chose face-to-face interaction with the teaching staff (57.47% vs 43.75%, 47.44%, and 49.28% for Q2, Q3, and Q4, respectively), while the same group missed interaction with other students the least (51.72% vs 70.00%, 62.82%, and 62.32% for Q2, Q3, and Q4, respectively).

## DISCUSSION

The analysis of the survey results revealed several important findings. First, despite a large amount of digital content, students overwhelmingly stated that the practical parts of the course (anatomical dissection, practical work on cadavers, prosections and models) were essential for learning the course material and could not be adequately transferred to an online environment. Second, students recognized online classes as a useful addition to contact classes, as well as stated that some segments of contact classes, such as traditional *ex cathedra* lectures and some seminars, could be completely or substantially replaced by online classes. Finally, the data acquired in this survey suggest that some differences in students’ responses could be related to how successful they were in learning the course material.

### Practical work remains a key component of a modern anatomical curriculum

To teach and assess such a difficult course as anatomy in medical education is both challenging and demanding. Different universities worldwide offer teaching approaches that suit their visions and teaching philosophies, spanning from extremely detail-oriented curricula to curricula devoted to problem solving and a general overview of the underlying structures. The best way of reaching these goals is yet to be determined, though many agree that a combination of teaching methods should be used, since none of the methods individually cover all the learning aims (21,22).

As the international debate on whether cadaver dissection is necessary in medical education continues, many educators still favor dissection over other tools (23) and it is still a preferred teaching method regardless of whether the anatomist is a “traditionalist” or a “modernist” (21). In our everyday work, we are challenged with cutting-edge visualization technologies, and debates have arisen whether dissection, as the gold standard in anatomy education, can and should be replaced (12). Our decision to continue with this traditional method is further supported by our students’ opinion that dissection is a fundamental tool for meeting the learning outcomes.

The data obtained in this survey clearly show that students consider anatomical dissection to be the most useful part of contact teaching. Support for anatomical dissection was expressed in some studies (24-26), while other studies called into question its overall impact on learning outcomes (14). During dissection, students directly study the three-dimensional topographic anatomy of the human body and discuss it in small groups with the aid of STAs and guidance of the teaching staff. Other types of practical work, such as demonstrations on cadavers, prosections and models, were also regarded as very useful.

After practical work, during online classes students mostly missed the interaction with STAs, followed by the interaction with other students. The fact that students evaluated their knowledge more easily during contact classes than during online classes supports the notion that through proper interaction, especially with peers and near-peers (27), students receive adequate feedback, which then better guides them while learning the course material. Such an interaction is vital for the absorption of the school’s hidden curriculum (28,29). In addition, over half of the students stated that they missed interaction with the teaching staff – this sentiment was more pronounced in the Q1 group than in the other groups. This discrepancy could be explained by the fact that the most successful students (Q1 group) had less difficulty learning the basic course material on their own; however, they needed additional explanations and feedback for more complex concepts, which falls outside the competence of STAs. Even though the teaching staff regularly posted answers and detailed explanations to students’ questions on Q&A forums on the Department’s e-learning platform, this did not compensate for the level of interaction achieved during contact classes.

Notably, students perceived as most useful the types of contact classes that are organized in small groups (up to 10 students per group), ie, practical classes involving dissection, demonstration, or self-guided practical work. The level of interaction between students, STAs, and teaching staff in such classes in our curriculum is typically very high. In contrast, students found traditional contact lectures to be the least useful type of contact classes – such lectures are held mostly *ex cathedra* in front of a large group of students (up to 150 students) and the level of interaction is typically very low. Finally, seminars, which are held in medium-sized groups (usually up to 25 students), were rated as more useful than lectures, but less useful than practical classes. This strongly suggests that, at least from the students’ perspective, the level of interaction greatly determines the usefulness of contact classes.

### Online teaching can greatly enhance traditional anatomical classes

Despite recognizing the importance of contact classes, especially those involving practical work, students also clearly identified the advantages of online learning and considered online classes a significant and useful addition to contact classes.

Our data show that contact lectures could almost entirely be transferred to an online environment since over 90% of students stated this and only 7% found classical lectures useful for learning the course material.

Students’ opinions diverged most when it came to seminars, with approximately half of the students considering seminars to be replaceable by online classes, while the other half considered seminars to be useful as contact classes. This can be explained by the level of interaction students experienced during contact seminars – students who achieved a higher interaction level with their teachers during seminars likely found them more useful than those who achieved a lower interaction level .

Students clearly stated that practical classes could in no way be replaced by online classes, which is in line with their sentiment that practical classes are the most useful part of contact classes.

Interestingly, students found pre-recorded video lectures to be the most useful segment of online learning, which is in contrast with their sentiment that lectures were the least useful type of contact classes. This can be explained by the following advantages of pre-recorded video lectures over contact lectures: they are constantly available through the e-learning platform and can be viewed at students’ leisure; students can re-view them multiple times, pause them, fast forward or rewind and re-watch parts they find more difficult to comprehend; this type of lectures also facilitates note taking or sketching of anatomical structures (30-33). The second most useful aspect of online classes for students were obligatory online quizzes, which were administered weekly and evaluated predominantly students’ comprehension and integration of the course material. Other studies support the positive effect of frequent quizzes on the final assessment in the anatomy course (34,35).

In general, it appears that students prefer classes with high interaction to be carried out as contact classes, while they find classes with low interaction better transferable to an online environment.

Nevertheless, certain segments of teaching deviated from this observation. Surprisingly, more students considered clinical cases more useful in the form of interactive online lessons (17%) than in the form of contact classes (13.5%), where clinical anatomy was discussed in small groups of five or six students. The fact that twice as many options were offered for the question on online classes as for the question on contact classes (12 options vs 7 options – on both question students could choose only three of the available options) clearly indicates that students consider clinical cases in an online format to be superior to those carried out through contact classes. This could be explained by the format of interactive lessons, which enables students to extensively interact with the course material via the user interface – thus allowing for a deeper understanding of the topic – even though there is no physical interaction with another person. The fact that students found active types of online content to be more useful (video lectures, quizzes, and quiz evaluations) than completely passive types of content (eg, presentation slides) shows that interaction is also important in online classes, albeit the way interaction is achieved in a virtual environment differs from the way it is achieved in a physical classroom.

Therefore, the goal of creating online teaching content should not be to simply replace contact classes that entail a low level of interaction. To the contrary, the online content that replaces contact classes should be organized in a way that it allows for greater interaction with the course material than it would be possible to achieve in equivalent contact classes. This is the reason why lectures and presentation segments of seminars are ideal for transfer into an online environment – these elements of contact classes are not only suitable for an online environment, but they offer significant benefits when presented in an online form, if done correctly. Online classes used in this way become a valuable asset in enhancing and enriching the curriculum and supporting contact classes.

Organizing online teaching as described has an additional benefit for contact classes – transferring suitable segments of contact classes to an online environment, where students watch them before contact classes, frees up time for other types of classes that students find more useful. Furthermore, conducting weekly quizzes in an online format, rather than during practical classes as was previously done in our course, gives the teaching staff more time to establish meaningful interactions with students. Similarly, transferring clinical cases to interactive online lessons allows devoting more contact classes to anatomical dissection, while transferring lectures and presentation portions of seminars into pre-recorded video lectures (that students can view before arriving to contact classes) allows devoting more time in class to relevant discussions with students. Administrative requirements, such as keeping records of student test scores and attendance, are also more easily completed in an online environment. All of this enables the teaching staff and STAs to better focus on students’ needs, teaching, and practical work during contact classes.

### What affects students’ success in the course?

Among the more surprising findings of this survey is that only a quarter of first-year medical students considered the anatomy course too demanding, even though our Department maintained its traditional high demand when it came to anatomical detail-recognition and correct naming of anatomical structures, while simultaneously increasing the demands when it came to conceptual understanding of functional and clinical anatomy. Around 40% of the students considered the course to be adequately difficult, while a third of the students did not consider the course too demanding at all – this was even more pronounced in the Q1 group, where almost half of the students did not consider the course too demanding. This indicates that students recognize the importance of learning anatomy in detail, which is supported by the fact that 87% of students agreed or mostly agreed with the statement that the required course material is important for the medical profession (Question S16).

The large amount of detailed information that has to be memorized in our course demands that students possess good organization skills, as well as the ability of long-term planning of complex and time-consuming tasks. Research shows that the structural maturation of the prefrontal cortex extends well into the third decade of a human lifespan, which is connected to the functions of long-term planning, motivation, and directing emotions (36-40). This is in line with research showing that young adults nowadays reach psychosocial maturity at a later age than before (41,42). These facts should also be considered when discussing students’ success and perceptions of the course.

The data from our survey show that students from the Q4 group (the least successful on partial exams) reported having prepared significantly less for seminars and practical classes and that they also used the available online materials significantly less frequently. Furthermore, even though the differences between the time that students from different groups spent studying anatomy were not significant, students from the Q4 group spent the least time studying both during contact (11.3% less time than students from Q1 group) and online classes (14.5% less time than students from Q1 group). In general, the more successful a student group was on the exam, the more time these students spent studying anatomy, and *vice versa*.

Finally, student groups differed in the segments of teaching they found useful for learning the course material. The differences between the groups’ responses to the questions pertaining to contact classes were rather small. However, for some aspects of online classes these differences were substantial. Only half as many (around 20%) students from the Q4 group as students from other groups found additional explanations of challenging topics useful, while twice as many (around 40%) students from the Q4 group found links to animations and video materials useful. Furthermore, students from the Q4 group found online quizzes less useful than did students from other groups, while they found the evaluation of the online quizzes more useful than did other groups. The Q4 group was also the only group where a larger portion of students considered presentation slides as useful. All of this indicates that a larger proportion of students from the Q4 group had problems with learning the basic concepts, which is why they found the content that aids in this (presentation slides, links to animations, etc) more useful than the content that aids in acquiring a deeper understanding of the course material (quizzes, additional explanations). It is also possible that students from the Q4 groups had, on average, more problems determining priorities while studying and typically focused less on the topics being assessed in the exam. This is supported by the fact that the Q4 group was less successful on the partial exams, but also on the obligatory online quizzes, which they found less useful than other groups.

It should be noted that the results of our study are most applicable to anatomy courses with similar curricula, while certain aspects might not transfer as well to differently structured anatomy courses.

### Conclusions

In conclusion, the results of this survey suggest that: 1) practical classes, especially anatomical dissection, should stay a key component of a modern anatomical curriculum; 2) online teaching is a valuable asset to a modern anatomical curriculum and can greatly enhance traditional anatomical classes; 3) interaction (between students, with STAs, with teaching staff and with the course material itself) is vital for determining how students perceive classes and how effective classes are in aiding students in learning; 4) teaching staff should strive to encourage less successful students to enhance their perception of the course and the attention to the overall importance of effort, as well as to empower them for adaptive change.
