# Supplementary material for: The anatomy lesson of the SARS-CoV-2 pandemic: irreplaceable tradition (cadaver work) and new didactics of digital technology
Source: Croat Med J. 2021 Apr;62(2):173–86. doi: 10.3325/cmj.2021.62.173 (PMC8107989; doi:10.3325/cmj.2021.62.173)
Supplement: Supplementary material 1 [file CroatMedJ_62_s001.pdf]

Question	Responses					
I attended class regularly.	(1)	(2)	(3)	(4)	(5)	
I regularly used the available on-line material in learning.	(1)	(2)	(3)	(4)	(5)	
I consistently prepared for lectures during contact classes.	(1)	(2)	(3)	(4)	(5)	
I consistently prepared for seminars during contact classes.	(1)	(2)	(3)	(4)	(5)	
I consistently prepared for practicals during contact classes.	(1)	(2)	(3)	(4)	(5)	
I have put a great deal of effort into learning the required exam material.	(1)	(2)	(3)	(4)	(5)	
In this course, I have been challenged to learn more than I expected.	(1)	(2)	(3)	(4)	(5)	
The course was intellectually stimulating.	(1)	(2)	(3)	(4)	(5)	
I put the most time and effort into the preparation for the following partial written exam:	A1	A2	A3			
I put the least time and effort into the preparation for the following partial written exam:	A1	A2	A3			
I achieved my highest score on the following partial written exam:	A1	A2	A3			
I achieved my lowest score on the following partial written exam:	A1	A2	A3			
What final grade do you expect to earn in this course?	5 (A)	4 (B)	3 (C)	2 (D)	I do not expect a passing grade	Not sure
During contact classes I spent, on average, the following amount of hours per day studying Anatomy:						
During on-line classes I spent, on average, the following amount of hours per day studying Anatomy:						
In the academic year 2019/20 I spent, on average, the following amount of hours per day studying Anatomy:						
The lectures, seminars, and practicals complemented each other and were structured in a logical order.	(1)	(2)	(3)	(4)	(5)	
The course material (obligatory and supplementary textbooks and atlases) increased my knowledge and skills in the subject matter.	(1)	(2)	(3)	(4)	(5)	
The course material (additional readings and hand-outs on LMS) increased my knowledge and skills in the subject matter.	(1)	(2)	(3)	(4)	(5)	
The course workload and requirements were appropriate for the course level.	(1)	(2)	(3)	(4)	(5)	
The course was organized in a manner that helped me learn the exam material and understand the underlying anatomical concepts.	(1)	(2)	(3)	(4)	(5)	
Frequent quizzes (both during regular and on-line classes) contributed to my successfully mastering the required exam material.	(1)	(2)	(3)	(4)	(5)	
In general, the course was well organized.	(1)	(2)	(3)	(4)	(5)	
The official textbook covers a large portion of the exam material.	(1)	(2)	(3)	(4)	(5)	
The official textbook is well written and easy to understand, and it is easy to study from it and master the exam material.	(1)	(2)	(3)	(4)	(5)	
The official textbook has clear and well selected anatomical pitctures that facilitate the comprehension of the course material.	(1)	(2)	(3)	(4)	(5)	
The official textbook has well structured tables that facilitate the comprehension of the course material.	(1)	(2)	(3)	(4)	(5)	
Compared to the defined learning outcomes and exam material, the official textbook is: (Choose the answer that best reflects your opinion)	too extensive	not extensive enough	of appropriate extent	too extensive in some parts, and not extensive enough in others	I don't have an opinion on this topic	
What would be your overall grade for the official textbook:	(1)	(2)	(3)	(4)	(5)	
On-line material in the A1 block integrated well with the direct classes.	(1)	(2)	(3)	(4)	(5)	
On-line material in the A2a block integrated well with the direct classes.	(1)	(2)	(3)	(4)	(5)	
On-line classes are a significant and useful addition to direct/live classes, but can NOT replace them.	(1)	(2)	(3)	(4)	(5)	
On-line classes could completely replace all segments of contact classes (including practical work).	(1)	(2)	(3)	(4)	(5)	
On-line classes could only partially replace some segments of contact classes.	(1)	(2)	(3)	(4)	(5)	
On-line classes could NOT replace any segment of contact classes.	(1)	(2)	(3)	(4)	(5)	
Classes in this course (both contact and on-line) have helped me cover the course material.	(1)	(2)	(3)	(4)	(5)	
During contact classes, I could easily assess my knowledge and progress at any given time.	(1)	(2)	(3)	(4)	(5)	
During on-line classes, I could easily assess my knowledge and progress at any given time.	(1)	(2)	(3)	(4)	(5)	
Which segments of contact classes could be adequately (in full or almost in full) replaced with on-line classes? (You may choose multiple answers)	lectures	seminars	practical work (without dissection)	anatomical dissection	none	

Which segments of contact classes have you benefited the most from in preparing the course material? (You may choose up to 3 answers)	lectures	seminars	practical work (without dissection)	anatomical dissection	demonstrations (practical work with STAs)	clinical cases	nothing of the aforementioned				
Which segments of on-line classes have helped you most in covering the material? (You may choose up to 3 answers)	video lectures	self-assessment tests	mandatory on-line quizzes	option of evaluating on-line quizzes	presentation slides	hand-outs	clinical cases (in the form of interactive lessons)	textbook elaborations	additional explanations of challenging topics	link to animations and other video materials	forum
Which segments of contact classes did you miss the most during on-line classes? (You may choose up to 3 answers)	lectures	seminars	practical work (without dissection)	anatomical dissection	demonstrations (practical work with STAs)	clinical cases (in small groups)	none of the aforementioned				
Which of the following aspects of contact teaching did you miss the most during on-line classes to adequately prepare the material? (You may choose up to 3 answers)	face-to-face interaction with teaching staff	practical work	face-to-face interaction with STAs	face-to-face interaction with other students	none of the aforementioned						
Learning outcomes were clearly defined.	(1)	(2)	(3)	(4)	(5)						
Learning outcomes helped me learn the required course material.	(1)	(2)	(3)	(4)	(5)						
This course has increased my interest for the field of anatomy.	(1)	(2)	(3)	(4)	(5)						
I think the required course material is important for the medical profession.	(1)	(2)	(3)	(4)	(5)						
I think the required course material is important for my future professional work.	(1)	(2)	(3)	(4)	(5)						
It is clearly defined what students are expected to cover and know in the course.	(1)	(2)	(3)	(4)	(5)						
Students' knowledge is fairly and adequately evaluated during the course (in direct classes, partial exams, weekly quizzes).	(1)	(2)	(3)	(4)	(5)						
The quizzes and partial exams tested what was covered in the classes.	(1)	(2)	(3)	(4)	(5)						
Examination criteria are clearly defined in this course.	(1)	(2)	(3)	(4)	(5)						
The exam material is clearly defined in this course.	(1)	(2)	(3)	(4)	(5)						
The responsiveness of the teaching staff was appropriate.	(1)	(2)	(3)	(4)	(5)						
This course was too challenging.	(1)	(2)	(3)	(4)	(5)						
The STAs knew and were confident in the material related to this course.	(1)	(2)	(3)	(4)	(5)						
The STAs were adequately prepared for dissections.	(1)	(2)	(3)	(4)	(5)						
The STAs were clear in presenting subject matter and presented the material in an interesting and engaging way.	(1)	(2)	(3)	(4)	(5)						
I felt comfortable asking my STAs questions.	(1)	(2)	(3)	(4)	(5)						
The STAs were willing to answer questions and were able to answer questions clearly and completely.	(1)	(2)	(3)	(4)	(5)						
The STAs fostered intellectual communication among my peers.	(1)	(2)	(3)	(4)	(5)						
The STAs were able to adequately help students in their preparation for the practical examination.	(1)	(2)	(3)	(4)	(5)						
The STAs were well-prepared for each class they participated in.	(1)	(2)	(3)	(4)	(5)						
The STAs arrived to class on time.	(1)	(2)	(3)	(4)	(5)						
A sufficient number of STAs was present during practical work.	(1)	(2)	(3)	(4)	(5)						
Overall, the STAs performed well.	(1)	(2)	(3)	(4)	(5)						
Name three things in the course that helped you the most in studying and preparing for the exam.											
What were the positive aspects of this course?											
What were the negative aspects of this course?											
Name three aspects of the course that need improvement to help students better learn the required course material.											
