# Supplementary material for: The anatomy lesson of the SARS-CoV-2 pandemic: irreplaceable tradition (cadaver work) and new didactics of digital technology
Source: Croat Med J. 2021 Apr;62(2):173–86. doi: 10.3325/cmj.2021.62.173 (PMC8107989; doi:10.3325/cmj.2021.62.173)
Supplement: Supplementary table 1 [file CroatMedJ_62_s002.pdf]

Table S1. All questions from the survey that were analyzed in the study.

Questions in which students had to express how much they agreed with the statement by choosing a grade between 1 and 5.

		1	2	3	4	5	Mean grade	Median grade
**S1**	**I attended contact classes regularly.**	0.31%	0.61%	3.37%	19.94%	75.77%	**4.70**	**5**
**S2**	**I regularly used the available on-line material in learning.**	0.00%	3.37%	15.34%	33.44%	47.85%	**4.26**	**4**
**S3**	**I consistently prepared for lectures during contact classes.**	7.06%	17.79%	26.07%	31.60%	17.48%	**3.35**	**3**
**S4**	**I consistently prepared for seminars during contact classes.**	1.23%	6.75%	18.71%	38.96%	34.36%	**3.98**	**4**
**S5**	**I consistently prepared for practical work during contact classes.**	0.00%	2.15%	8.59%	35.89%	53.37%	**4.40**	**5**
**S6**	**I have put a great deal of effort into learning the required exam material.**	0.00%	1.23%	7.98%	24.54%	66.26%	**4.56**	**5**
**S7**	**Frequent quizzes (both during regular and on-line classes) contributed to my successfully mastering the required exam material.**	3.68%	6.44%	11.66%	24.23%	53.99%	**4.18**	**5**
**S8**	**On-line classes are a significant and useful addition to contact classes but cannot replace them.**	1.84%	4.91%	13.50%	28.83%	50.92%	**4.22**	**5**
**S9**	**On-line classes could completely replace all segments of contact classes (including practical work).**	44.79%	27.91%	13.19%	8.59%	5.52%	**2.02**	**2**
**S10**	**On-line classes could only partially replace some segments of contact classes.**	4.91%	8.59%	16.87%	31.90%	37.73%	**3.89**	**4**
**S11**	**On-line classes could NOT replace any segment of contact classes.**	39.57%	20.86%	16.56%	11.96%	11.04%	**2.34**	**2**
**S12**	**Classes in this course (both contact and on-line) helped me cover the course material.**	1.84%	6.75%	16.87%	33.44%	41.10%	**4.05**	**4**
**S13**	**During contact classes, I could easily assess my knowledge and progress at any given time.**	1.23%	4.91%	20.25%	36.20%	37.42%	**4.04**	**4**
**S14**	**During on-line classes, I could easily assess my knowledge and progress at any given time.**	6.75%	21.17%	32.52%	26.99%	12.58%	**3.17**	**3**
**S15**	**Learning outcomes helped me learn the required course material.**	3.68%	11.04%	21.78%	30.37%	33.13%	**3.78**	**4**
**S16**	**I think the required course material is important for the medical profession.**	0.31%	1.23%	11.35%	30.06%	57.06%	**4.42**	**5**
**S17**	**The responsiveness of the teaching staff was appropriate.**	0.31%	2.15%	7.98%	20.86%	68.71%	**4.56**	**5**
**S18**	**This course was too challenging.**	9.20%	25.46%	40.18%	21.78%	3.37%	**2.85**	**3**
**S19**	**Overall, the STAs performed well.**	0.00%	0.31%	5.52%	19.02%	75.15%	**4.69**	**5**

Questions in which students had to write a number as a response

**S20**	**During contact classes I spent, on average, the following amount of hours per day studying Anatomy:**	**4.06 ± 1.52**
**S21**	**During on-line classes I spent, on average, the following amount of hours per day studying Anatomy:**	**5.56 ± 2.46**
**S22**	**In the academic year 2019/20 I spent, on average, the following amount of hours per day studying Anatomy:**	**4.86 ± 1.78**

Questions in which students could choose one or more answers from a predefined list

**S23**	**Which segments of contact classes could be adequately (in full or almost in full) replaced with on-line classes? (You may choose multiple answers)**	**lectures**	**seminars**	**practical work (without dissection)**	**anatomical dissection**	**none**		
		91.41%	47.55%	6.75%	0.92%	7.06%		
**S24**	**Which segments of contact classes have you benefited the most from in preparing the course material? (You may choose up to 3 answers)**	**lectures**	**seminars**	**practical work (without dissection)**	**anatomical dissection**	**demonstrations (practical work with STAs)**	**clinical cases**	**nothing of the aforementioned**
		7.36%	42.33%	46.93%	81.90%	69.33%	13.50%	1.23%
**S25**	**Which segments of on-line classes have helped you most in covering the material? (You may choose up to 3 answers)**	**video lectures**	**self-assessment tests**	**mandatory on-line quizzes**	**option of evaluating on-line quizzes**	**presentation slides**	**hand-outs**	**clinical cases (in the form of interactive lessons)**
		60.74%	14.42%	54.29%	30.37%	6.44%	48.47%	16.87%
		**textbook elaborations**	**additional explanations of challenging topics**	**link to animations and other video materials**	**forum**	**nothing of the aforementioned**		
		27.91%	37.42%	26.07%	3.37%	1.23%		
**S26**	**Which segments of contact classes did you miss the most during on-line classes? (You may choose up to 3 answers)**	**lectures**	**seminars**	**practical work (without dissection)**	**anatomical dissection**	**demonstrations (practical work with STAs)**	**clinical cases (in small groups)**	**none of the aforementioned**
		3.07%	24.54%	49.08%	81.90%	65.64%	5.21%	3.99%
**S27**	**Which of the following aspects of contact teaching did you miss the most during on-line classes to adequately prepare the material? (You may choose up to 3 answers)**	**face-to-face interaction with teaching staff**	**practical work**	**face-to-face interaction with STAs**	**face-to-face interaction with other students**	**none of the aforementioned**		
		51.11%	81.60%	72.09%	60.74%	3.07%		
