# Supplementary material for: The anatomy lesson of the SARS-CoV-2 pandemic: irreplaceable tradition (cadaver work) and new didactics of digital technology
Source: Croat Med J. 2021 Apr;62(2):173–86. doi: 10.3325/cmj.2021.62.173 (PMC8107989; doi:10.3325/cmj.2021.62.173)
Supplement: Supplementary table 2 [file CroatMedJ_62_s003.pdf]

Table S2. Instructions for choosing responses to questions in which students had to assess their agreement with a particular statement

**Grade 1** means that you **completely disagree** with the statement.
**Grade 2** means that you **mostly disagree** with the statement.
**Grade 3** means that you **neither agree nor disagree** with the statement.
**Grade 4** means that you **mostly agree** with the statement.
**Grade 5** means that you **completely agree** with the statement.
