# Supplementary material for: The anatomy lesson of the SARS-CoV-2 pandemic: irreplaceable tradition (cadaver work) and new didactics of digital technology
Source: Croat Med J. 2021 Apr;62(2):173–86. doi: 10.3325/cmj.2021.62.173 (PMC8107989; doi:10.3325/cmj.2021.62.173)

Supplementary Figure 1 – Bar graphs showing the results to the questions in which students had to pick answers from a predefined list. Student response frequencies are shown for the following questions: (**A**) which segments of on-line classes helped students the most in preparing the course material (Question S25), (**B**) which types of contact classes students missed most during on-line classes (Question S26) and (**C**) which aspects of contact classes students missed the most during on-line classes (Question S27).

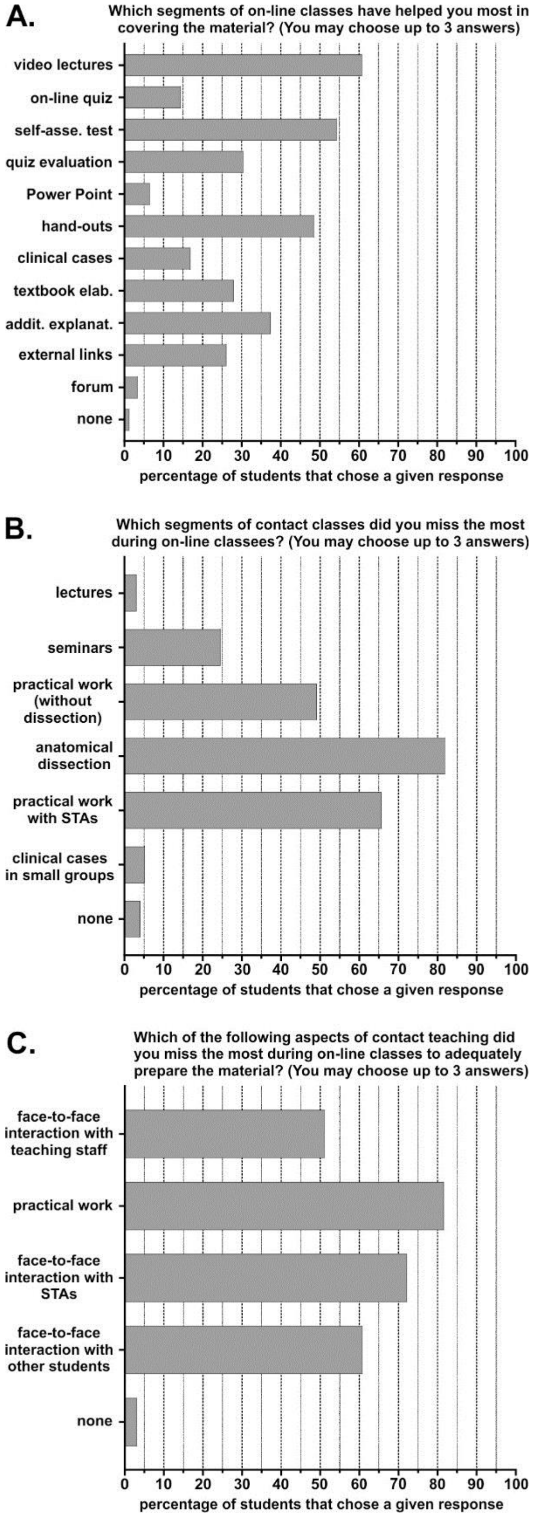

Supplement: Supplementary figure 1 [file CroatMedJ_62_s004.pdf]
