# Supplementary material for: The anatomy lesson of the SARS-CoV-2 pandemic: irreplaceable tradition (cadaver work) and new didactics of digital technology
Source: Croat Med J. 2021 Apr;62(2):173–86. doi: 10.3325/cmj.2021.62.173 (PMC8107989; doi:10.3325/cmj.2021.62.173)

Supplementary Figure 2 – Violin plots showing a smoothed distribution of student responses from different groups (divided into quartiles based on their written partial exam score during continuous assessment: Q1, Q2, Q3 and Q4) to questions in which statistically significant differences between groups were found. The dotted line in the violin plots represents the median of the responses to each question. The size of the bulge in a violin plot is proportional to the frequency of students that chose the grade displayed on the y-axis. Statistically significant differences are marked on the plots (Kruskal–Wallis test with the Dunn post hoc test).. The different panels show responses to the question pertaining to (**A**) preparation for lectures (Question S3), (**B**) preparation for seminars (Question S4) and (**C**) preparation for practical classes (Question S5).

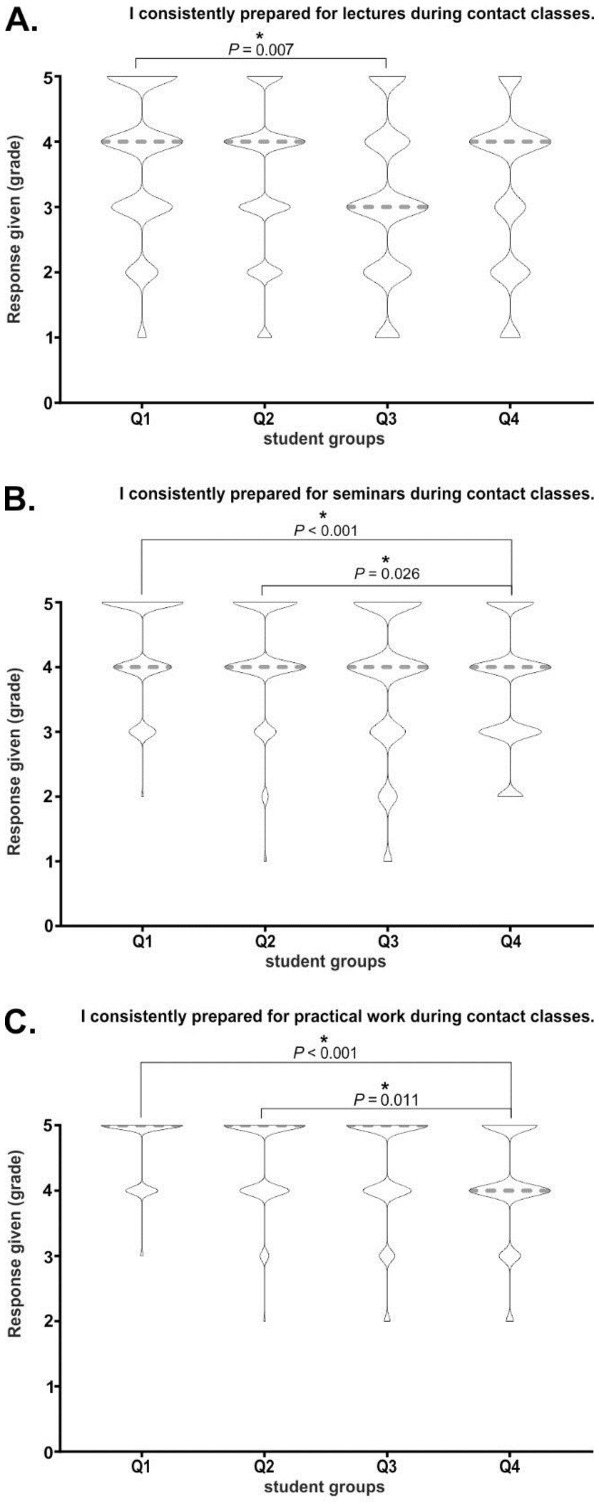

Supplement: Supplementary figure 2 [file CroatMedJ_62_s005.pdf]
