# Supplementary material for: The anatomy lesson of the SARS-CoV-2 pandemic: irreplaceable tradition (cadaver work) and new didactics of digital technology
Source: Croat Med J. 2021 Apr;62(2):173–86. doi: 10.3325/cmj.2021.62.173 (PMC8107989; doi:10.3325/cmj.2021.62.173)

Supplementary Figure 3 – Differences between student groups (divided into quartiles based on their written partial exam score during continuous assessment: Q1, Q2, Q3 and Q4) in responses given to the questions pertaining to the number of hours spent studying during contact and on-line classes. The markers represent the difference between group means and the horizontal bars represent the 95% confidence interval (CI) for the difference between the group means calculated from Tukey's post-hoc test. The vertical dotted line represents a difference of 0 (no difference); if the 95% CI crosses the dotted line, the difference is not statistically significant. (**A**) Differences between groups means for the question pertaining to the number of hours spent studying Anatomy during contact classes (Question 8). (**B**) Differences between groups means for the question pertaining to the number of hours spent studying Anatomy during on-line classes (Question 9).

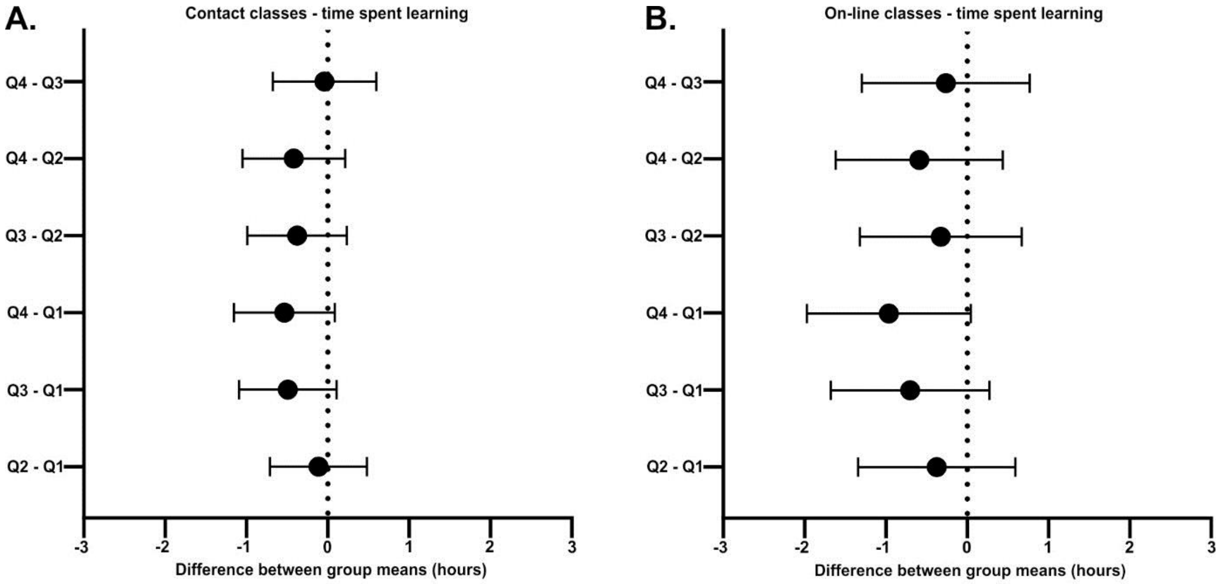

Supplement: Supplementary figure 3 [file CroatMedJ_62_s006.pdf]
